# P-340. Evaluating the Efficacy of Nasal Iodophor Antisepsis in Reducing *Candida auris* Nasal Carriage in Nursing Home Residents

**DOI:** 10.1093/ofid/ofae631.542

**Published:** 2025-01-29

**Authors:** Gabrielle Gussin, Raveena D Singh, Raheeb Saavedra, Eric Nguyen, Julie A Shimabukuro, Cassiana E Bittencourt, Susan Huang

**Affiliations:** University of California, Irvine School of Medicine, Division of Infectious Diseases, Irvine, California; University of California, Irvine School of Medicine, Division of Infectious Diseases, Irvine, California; University of California, Irvine School of Medicine, Division of Infectious Diseases, Irvine, California; University of California, Irvine, Irvine, California; University of California, Irvine Health, Orange, California; University of California, Irvine Health, Orange, California; University of California, Irvine School of Medicine, Irvine, California

## Abstract

**Background:**

*Candida auris* is an emerging drug-resistant yeast that is spreading rapidly in U.S. healthcare facilities, particularly in nursing homes. Like MRSA, *C. auris* is commonly found in the nose, raising questions about whether nasal iodophor application can reduce carriage.

**Figure:**
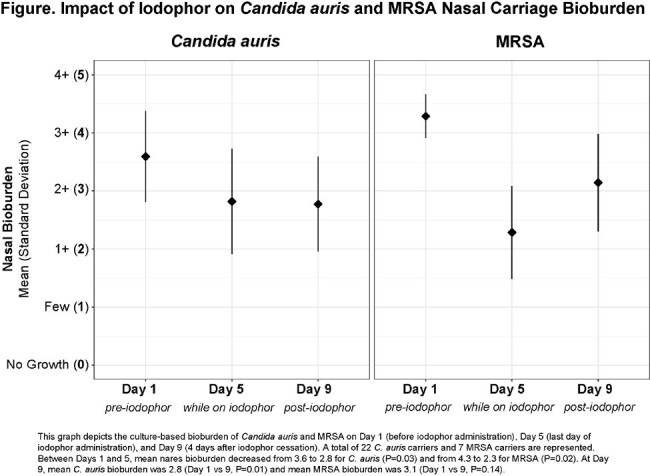

**Methods:**

We evaluated the impact of iodophor on *C. auris* nasal carriage at two nursing homes. Nasal carriers were identified from point prevalence sampling of bilateral nares of residents for both *C. auris* and MRSA conducted at each nursing home.

Nasal iodophor was given to carriers according to a 5-day twice-daily decolonization protocol. Nasal swabs for *C. auris* and MRSA culture were collected on Day 1 (pre-iodophor), Day 5 (last day of iodophor administration), and Day 9 (4 days after iodophor cessation). Bioburden was assessed on an ordinal scale (none = 0, few =1, 1+ = 2, 2+ = 3, 3+ = 4, and 4+ = 5). Paired t-tests compared *C. auris* and MRSA bioburden between timepoints.

**Results:**

Twenty-two residents with *C. auris* nares carriage completed the evaluation. Seven (32%) also harbored MRSA. Between Days 1 and 5, mean nares bioburden decreased from 3.6 to 2.8 for *C. auris* (P=0.03) and from 4.3 to 2.3 for MRSA (P=0.02) (**Figure**). Comparing Day 9 to Day 1, mean *C. auris* bioburden at Day 9 was 2.8 (P=0.01) and mean MRSA bioburden at Day 9 was 3.1 (P=0.14).

**Conclusion:**

Five days of nasal iodophor antisepsis appeared to suppress both *C. auris* and MRSA carriage. Rebound growth four days after iodophor discontinuation was seen for MRSA but not *C. auris*, although numbers were small. Nasal iodophor may be an effective strategy for reducing nasal carriage in high-risk settings. The value of repeated antisepsis for sustained bioburden reduction was not evaluated in this study.

**Disclosures:**

**Raveena D. Singh, MA**, Xttrium Laboratories: Conducting studies in which participating hospital patients received contributed antiseptic products outside the submitted work **Raheeb Saavedra, AS**, Xttrium Laboratories: Conducting studies in which participating hospital patients received contributed antiseptic products outside the submitted work **Susan Huang, MD, MPH**, Xttrium Laboratories: Conducting studies in which participating hospital patients received contributed antiseptic products outside the submitted work

